# Supplementary material for: Anti-Invasion and Antiangiogenic Effects of Stellettin B through Inhibition of the Akt/Girdin Signaling Pathway and VEGF in Glioblastoma Cells
Source: Cancers (Basel). 2019 Feb 14;11(2):220. doi: 10.3390/cancers11020220 (PMC6406657; doi:10.3390/cancers11020220)
Supplement: Supplementary file 1 [file cancers-11-00220-s001.pdf]

Supplementary Materials: Anti-Invasion and Antiangiogenic Effects of Stellettin B through Inhibition of the Akt/Girdin Signaling Pathway and VEGF in Glioblastoma Cells

Shu-Yu Cheng, Nan-Fu Chen, Pi-Yu Lin, Jui-Hsin Su, Bing-Hung Chen, Hsiao-Mei Kuo, Chun-Sung Sung, Ping-Jyun Sung, Zhi-Hong Wen and Wu-Fu Chen

Table S1. Zebrafish survival rate.

Stellettin B (nM)	Total number	Death number	Death rate (%)
0	18	0	0
10	18	0	0
50	18	0	0
100	18	1	5.56
250	18	2	11.11

© 2019 by the authors. Licensee MDPI, Basel, Switzerland. This article is an open access article distributed under the terms and conditions of the Creative Commons Attribution (CC BY) license (http://creativecommons.org/licenses/by/4.0/).
